# Supplementary material for: Fertilization-induced synergid cell death by RALF12-triggered ROS production and ethylene signaling
Source: Nat Commun. 2025 Mar 29;16:3059. doi: 10.1038/s41467-025-58246-y (PMC11953305; doi:10.1038/s41467-025-58246-y)
Supplement: Supplementary file 1 — Supplementary Information [file 41467_2025_58246_MOESM1_ESM.pdf]

**SUPPLEMENTARY INFORMATION**

# Fertilization-induced synergid cell death by RALF12-triggered ROS production and ethylene signaling

Junyi Chen[1,*], Huan Wang[1,*], Jinlin Wang[1], Xixi Zheng[2], Wantong Qu[1], Huijian Fang[1], Shuang Wang[1], Le He[1], Shuang Hao[1] and Thomas Dresselhaus[2]

[1]Hubei Key Laboratory of Genetic Regulation and Integrative Biology, School of Life Sciences, Central China Normal University, Wuhan, Hubei Province, China.
[2]Cell Biology and Plant Biochemistry, University of Regensburg, Regensburg, Germany.
[*]These authors contributed equally: Junyi Chen, Huan Wang.

correspondence: junyi.chen@ccnu.edu.cn; thomas.dresselhaus@ur.de

## Supplementary Information

Supplementary Fig. 1: Timing of synergid cell death during the fertilization process in maize.

Supplementary Fig. 2: RNA-seq analysis at precise synergid degenerating stages.

Supplementary Fig. 3: The major transcriptional activation/repression wave in the persistent synergid cell occurs about 24 HAP.

Supplementary Fig. 4: *In planta* activation of synergid-specific gene promoters by ZmMYB98 and secretion of gene products.

Supplementary Fig. 5: ZmRALF12 interacts with co-expressed ZmFERL1, ZmFERL16 and ZmLLG3 receptors.

Supplementary Fig. 6: Loss-of-function *zmralf12-/-* mutation after CRISPR-Cas9-editing.

Supplementary Fig. 7: ZmRALF12 specifically triggers high levels of ROS production and accumulation alongside the filiform apparatus of synergid cells.

Supplementary Fig. 8: ZmRALF12-triggered mitochondrial ROS production is RBOH- and $Ca^{2+}$ channel-dependent.

Supplementary Fig. 9: ZmRALF12 triggers granular ROS accumulation at the filiform apparatus adjacent region in a time- and concentration-dependent manner.

Supplementary Fig. 10: Peroxisomal function-related genes are significantly activated indicating an oxidative stress status in synergids after 18 HAP.

Supplementary Fig. 11: Genes encoding $Ca^{2+}$ channels, $Ca^{2+}$ sensors, and $Ca^{2+}$ signaling components involved in senescence and cell death regulation are activated in synergids after 18 HAP.

Supplementary Fig. 12: Autophagy activation and subsequent elimination of the persistent synergid cell through synergid-endosperm (SE) fusion.

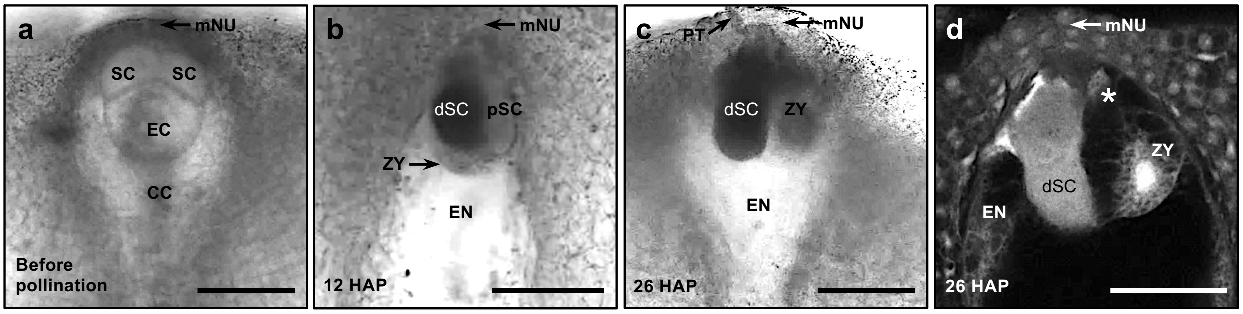


**Supplementary Fig. 1 Timing of synergid cell death during the fertilization process in maize.** **a**–**c** Bright-field micrographs showing the embedded female gametophyte (embryo sac) containing two synergid cells before fertilization (a), at 12 HAP (this corresponds to about 4 HAF) (b) and at 26 HAP (about 18 HAF), respectively. The receptive synergid invaded by a pollen tube appears dark beside the persistent synergid cell and zygote, respectively. **d** Representative CLSM image of a fertilized embryo sac at 26 HAP. The persistent synergid is no longer visible, leaving the degenerated receptive synergid and the zygote at the micropylar region of the embryo sac. Observation was performed with maize B73 inbred line. Experiments were repeated three times with similar results. Asterisk marks residues of the disappeared persistent synergid cell. Abbreviations: CC, central cell; dSC, degenerated receptive synergid cell; EC, egg cell; EN, endosperm; HAP, hours after pollination; HAF, hours after fertilization; mNU, micropylar nucellus; pSC, persistent synergid cell; PT, pollen tube; SC, Synergid cell; ZY, zygote. Scale bars, 50 μm.

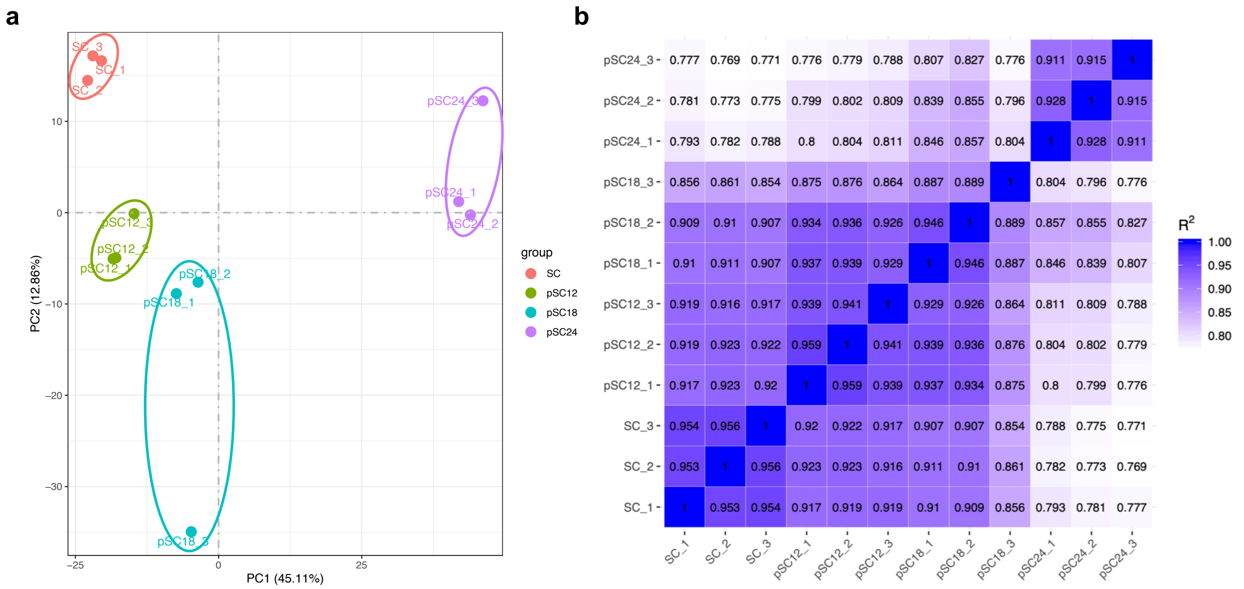


**Supplementary Fig. 2 RNA-seq analysis at precise synergid degenerating stages. a** Principal component analysis (PCA) of synergid cell RNA-seq data at indicated stages. **b** RNA-seq data of the same synergid stage are highly correlated. The correlation coefficients between different samples were calculated according to Pearson's correlation coefficient method. Abbreviations: SC, synergid cell before pollination; pSC12, persistent synergid cell at 12 hours after pollination (HAP); pSC18, persistent synergid cell at 18 HAP; pSC24, persistent synergid cell at 24 HAP.

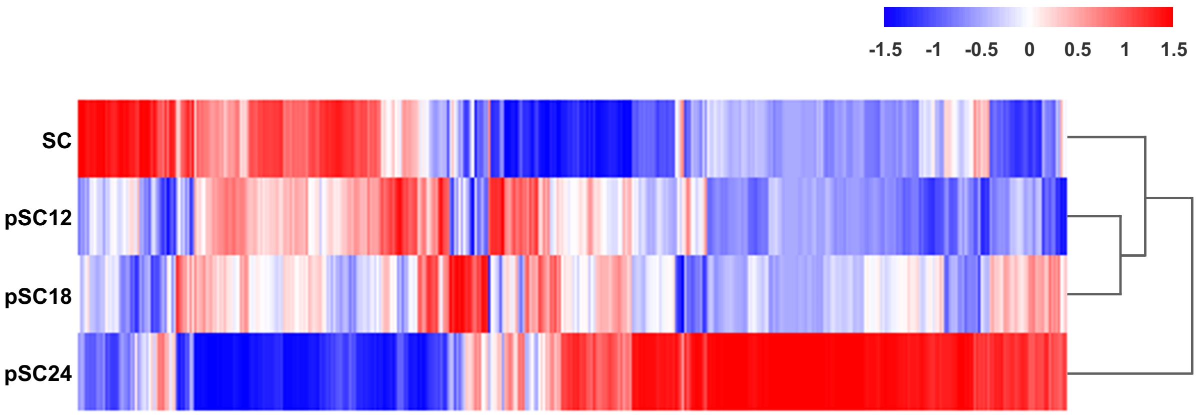


**Supplementary Fig. 3 The major transcriptional activation/repression wave in the persistent synergid cell occurs about 24 HAP.** Heatmap showing the expression pattern of differentially expressed genes (DEGs) in synergid cells across precise stages after fertilization and during synergid degeneration. Genes with abs($\log_2$FC) > 1 and adjusted $P < 0.05$ in at least one synergid cell degenerating stage comparison are included. For abbreviations see Supplementary Fig. 2.

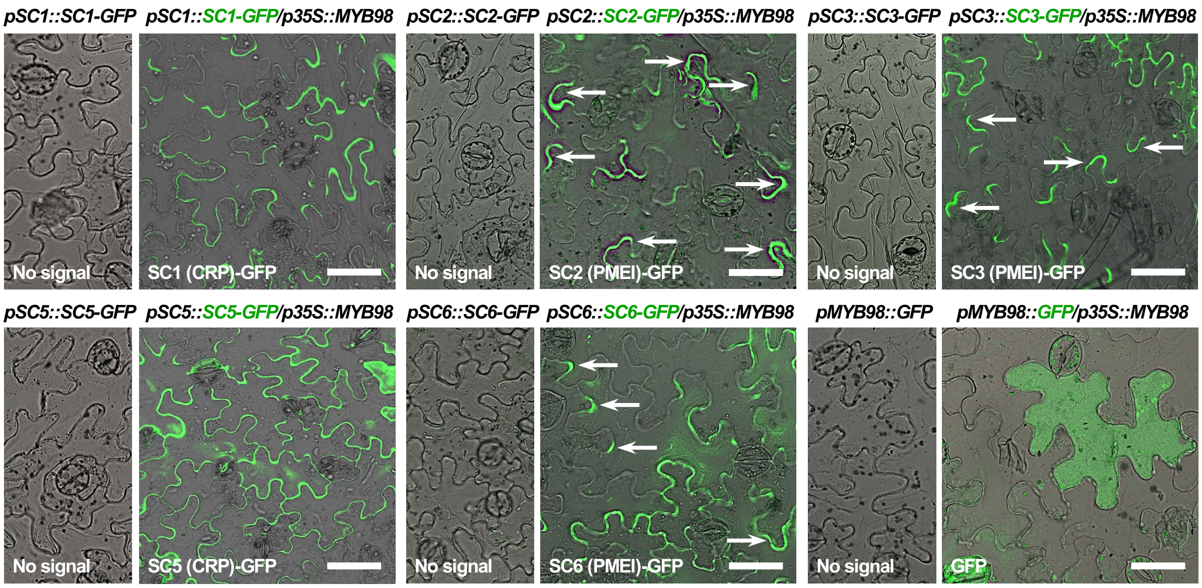


**Supplementary Fig. 4 *In planta* activation of synergid-specific gene promoters by ZmMYB98 and secretion of gene products.** *N. benthamiana* leaves were infiltrated with indicated constructs. *pMYB98::GFP* was used as a control. The most highly expressed synergid-specific gene products were secreted to the extracellular space. Arrows indicate the strongest signals at the lobe tips of pavement cells. Scale bars, 50 μm. Experiments were repeated three times with similar results.

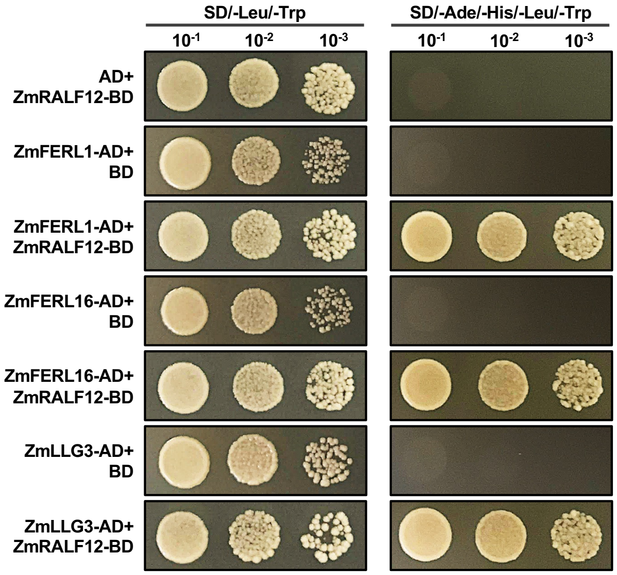


**Supplementary Fig. 5 ZmRALF12 interacts with co-expressed ZmFERL1, ZmFERL16 and ZmLLG3 receptors.** Yeast two-hybrid (Y2H) assay showing interactions and controls in indicated media. Experiments were repeated three times with consistent results. Source data are provided as a Source Data file. AD, GAL4 activation domain; BD, GAL4 DNA-binding domain. SD, synthetic defined medium.

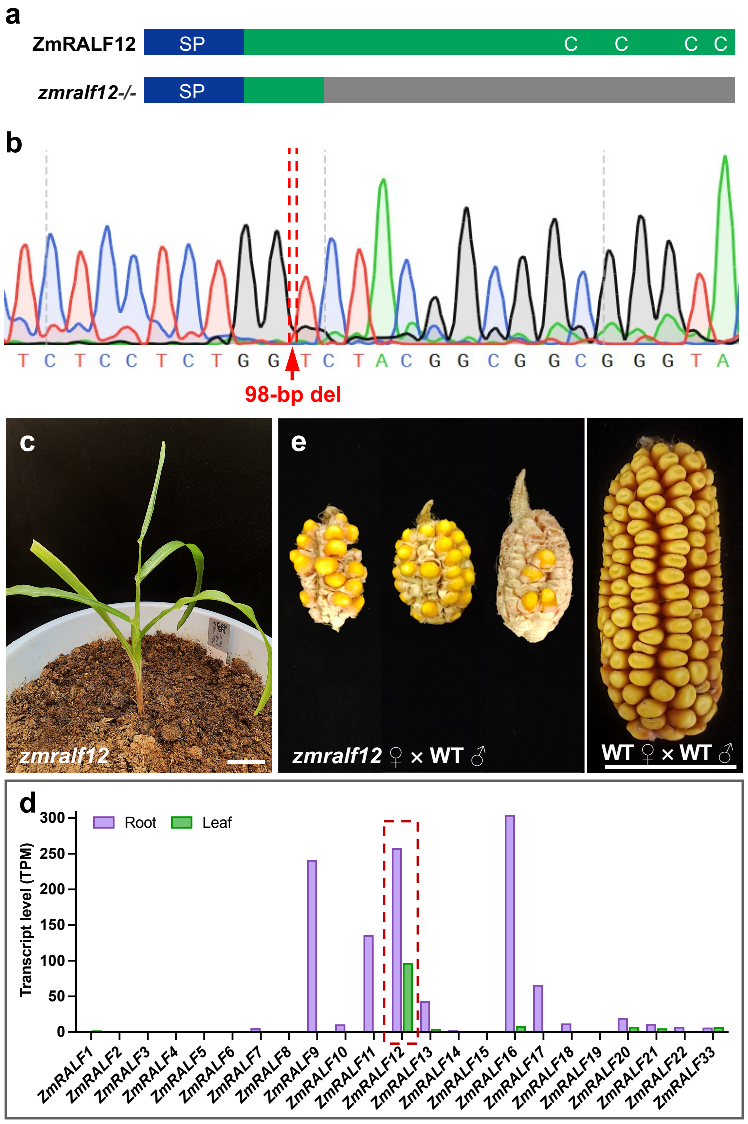


**Supplementary Fig. 6 Generation of a loss-of-function *zmralf12-/-* mutation after CRISPR-Cas9-editing**. **a** Schematic diagram of protein structure of wild-type ZmRALF12 and the CRISPR-Cas9-edited *zmralf12-/-* mutant. SP, signal peptide; C, conserved cysteine residues. Gray box indicates missense sequence due to fragment deletion. **b** Sequencing result of the mutation site of *ZmRALF12* in the *zmralf12-/-* mutant. Red arrow indicates deleted base pairs. **c** *zmralf12* mutant plants showed severe growth retardation and a dwarf phenotype. **d** Gene expression analysis of maize *RALF* family members in root and leaf tissues. *ZmRALF12* has moderate expression in maize roots and leaves. Transcript levels are provided in transcripts per million (TPM). **e** *zmralf12* mutant plants produced small cobs with limited number of ovaries and kernels. Source data are provided as a Source Data file. Scale bars, 5 cm.

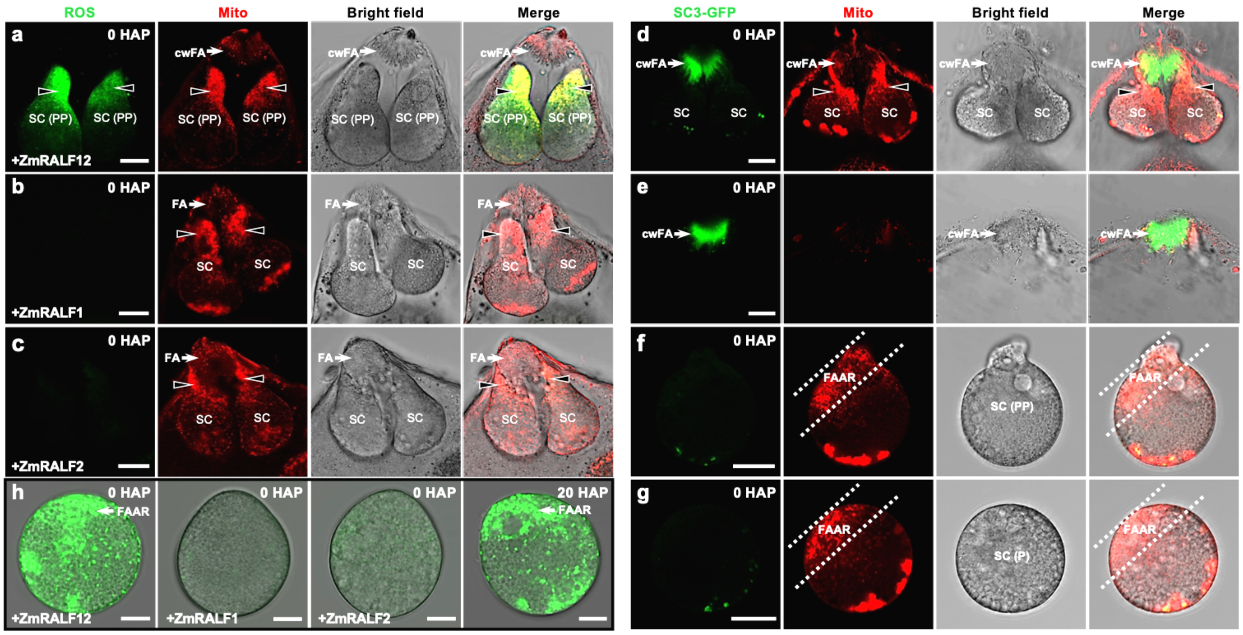


**Supplementary Fig. 7 ZmRALF12 specifically triggers high levels of ROS production and accumulation alongside the filiform apparatus of synergid cells. a–c** Ovule cultivation assay showing that ZmRALF12 (a), but not ZmRALF1 (b) nor ZmRALF2 (c) induce ROS production and accumulation alongside the FA region in synergid cells. **d** Mitochondria constitutively accumulate alongside the filiform apparatus (FA) adjacent region (FAAR) of synergid cells (black arrowheads). The thickened cell wall of FA (cwFA) is shown using *pZmSC3::ZmSC3-GFP* as a marker. **e–g** The mitochondria-enriched region is maintained in synergid protoplasts after manual isolation. During microdissection, synergid protoplast was detached from the cwFA (e, f). The mitochondria accumulating region at the micropylar pole was preserved in synergid protoplasts even after one hour (g). **h** ZmRALF12 application (4 μM for 30 min) specifically triggers strong and uneven ROS accumulation at the FAAR of dissected synergid cells, comparable to persistent synergid cells at 20 HAP. Experiments were repeated three times with similar results and presented are representative images. Abbreviations: cwFA, cell wall of filiform apparatus. FA, filiform apparatus; FAAR, filiform apparatus adjacent region in dissected synergid protoplast; SC, synergid cell. SC (P), synergid cell protoplast. SC (PP), synergid cell pre-protoplast. Scale bars, 20 μm (a–g) and 10 μm (h), respectively.

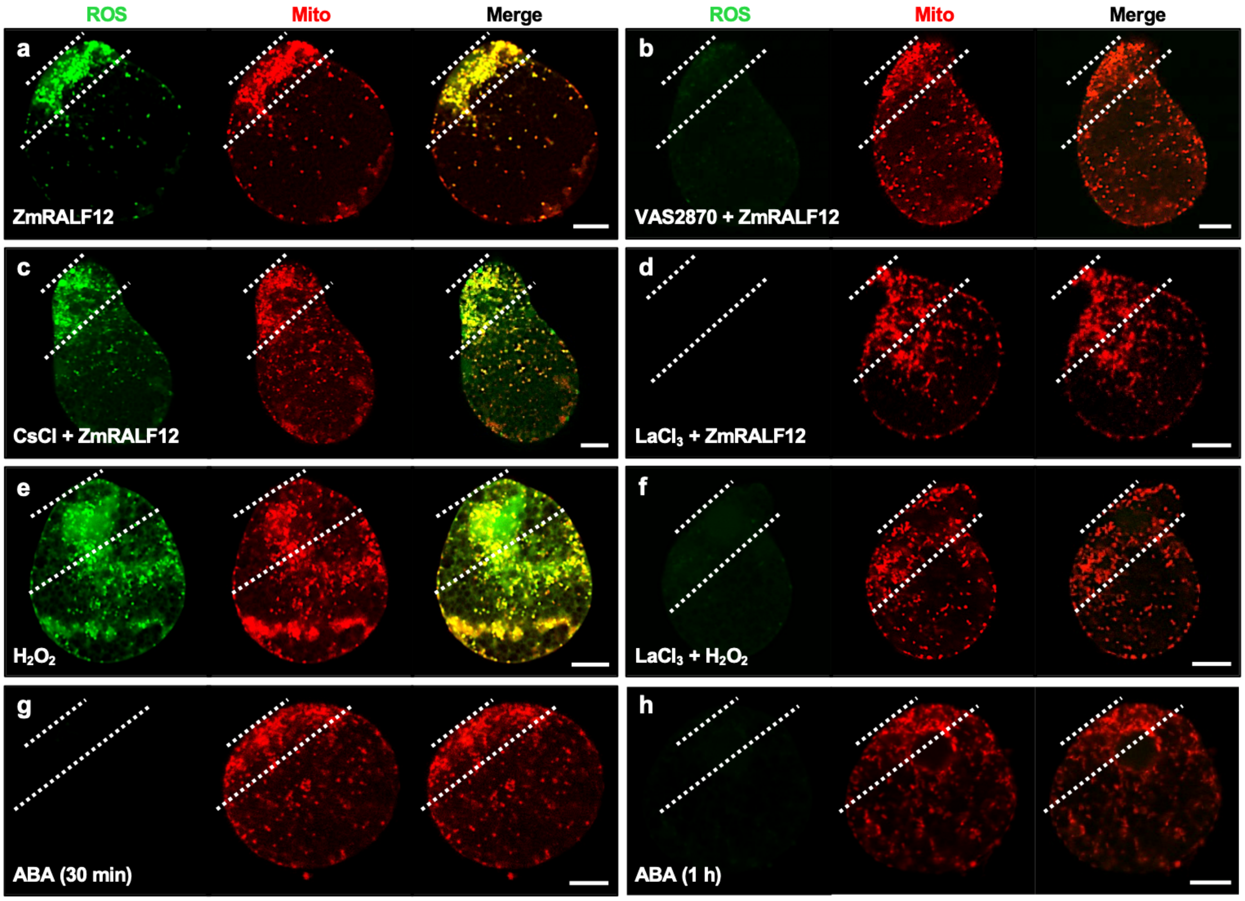


**Supplementary Fig. 8 ZmRALF12-triggered mitochondrial ROS production is RBOH- and $Ca^{2+}$ channel-dependent. a** ZmRALF12 application (4 μM for 30 min) triggers mitochondrial ROS production in synergid cells. **b** ZmRALF12-triggered ROS production in synergid mitochondria was lost after treatment with the RBOH inhibitor VAS2870. **c**,**d** $Ca^{2+}$ channel inhibitor ($LaCl_3$), but not $K^+$ channel inhibitor (CsCl) leads to loss of RALF12-triggered mitochondrial ROS production. **e**,**f** $H_2O_2$-induced ROS accumulation in synergid mitochondria is lost after additional $Ca^{2+}$ channel inhibitor ($LaCl_3$) application. **g**,**h** ABA treatment (20 μM for 30 min or 1 h) does not trigger mitochondrial ROS production in synergid cells. Dashed lines show the filiform apparatus adjacent region (FAAR) exhibiting accumulated mitochondria distribution. Scale bars, 10 μm. Experiments in a–f were repeated three times with similar results. ABA treatment was performed two times with similar results.

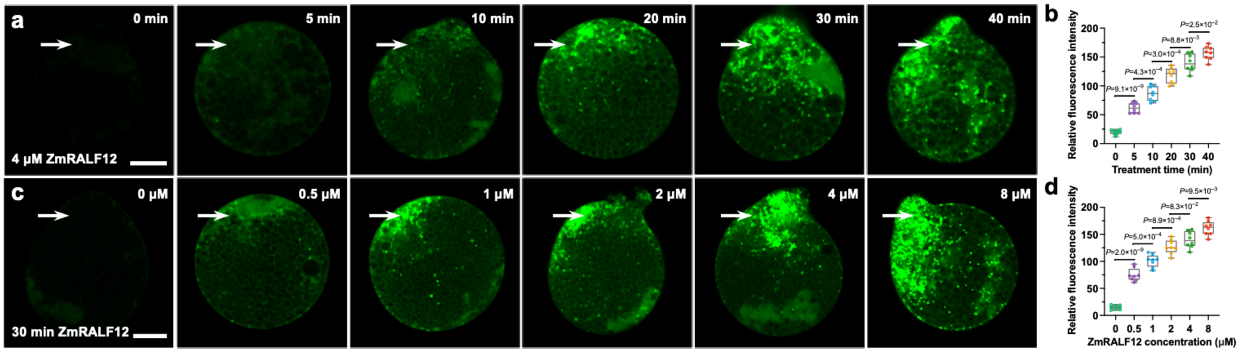


**Supplementary Fig. 9 ZmRALF12 triggers granular ROS accumulation at the filiform apparatus adjacent region in a time- and concentration-dependent manner.** Synergid cells for ZmRALF12 treatment were manually isolated from unfertilized ovules. **a** ZmRALF12 treatment induces granular ROS accumulation at the filiform apparatus adjacent region (FAAR) of synergid cells (arrows) ultimately expanding within the whole cell. **b** Quantification of ROS fluorescence intensity at the FAAR after ZmRALF12 (4 μM) application at indicated time points (n=8). **c** Increasing concentrations of ZmRALF12 significantly increases granular ROS accumulation. **d** Quantification of ROS fluorescence intensity at the FAAR after ZmRALF12 (30 min) application with indicated concentrations (n=8). Arrows indicate FAAR. Data for relative ROS fluorescence intensity at the FAAR are present in box-and-whisker plots. Bottom and top of the box, 25th and 75th percentiles; centre line, 50th percentile; whiskers, minimum and maximum data. Statistical significance is determined by two-tailed Student's *t*-test. Source data are provided as a Source Data file. Scale bars, 10 μm.

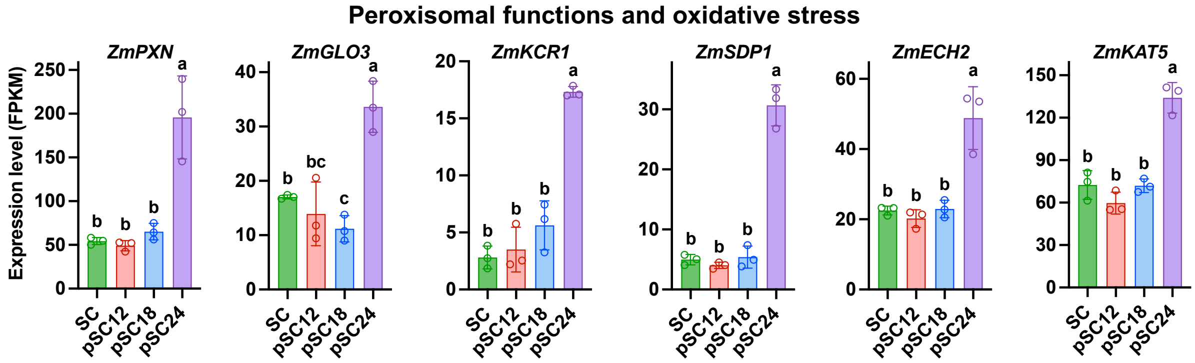


**Supplementary Fig. 10 Peroxisomal function-related genes are significantly activated indicating an oxidative stress status in synergids after 18 HAP.** Expression analysis of selected maize genes involved in peroxisomal functions including NAD$^+$ import (*ZmPXN*), 2-hydroxyacid oxidation (*ZmGLO3*), very long-chain fatty acids (VLCFAs) synthesis for peroxisomal metabolization (*ZmKCR1*) and fatty acid oxidation (*ZmSDP1*, *ZmECH2*, and *ZmKAT5*). Data are presented as mean values ± SD. For statistical analysis, count data at the gene level were analyzed with DESeq2[64]. Stage-to-stage comparisons were performed and corrected for multiple testing over all genes and cell stage comparisons using false discovery rate (FDR). Letters above bars indicate significant differences (adjusted $P < 0.05$). Source data and adjusted $P$ values are provided in the source data file. The expression pattern indicates an oxidative damage status in persistent synergid cells after 18 HAP.

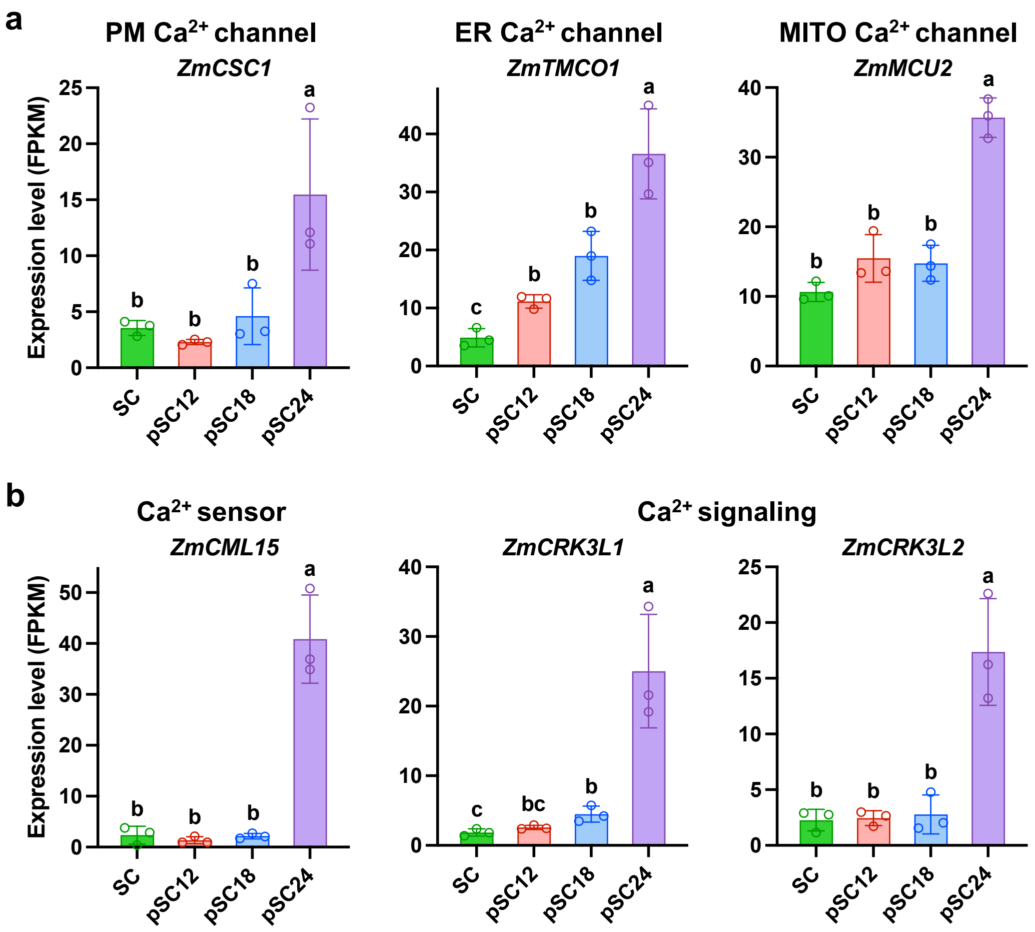


**Supplementary Fig. 11 Genes encoding $Ca^{2+}$ channels, $Ca^{2+}$ sensors, and $Ca^{2+}$ signaling components involved in senescence and cell death regulation are activated in synergids after 18 HAP. a** Activation of genes encoding a plasma membrane (PM) $Ca^{2+}$ permeable stress-gated cation channel (*ZmCSC1*), an ER membrane $Ca^{2+}$ load-activated $Ca^{2+}$ channel (*ZmTMCO1*), and a mitochondrial (MITO) inner membrane $Ca^{2+}$ uniporter (*ZmMCU2*) that mediates $Ca^{2+}$ uptake into mitochondria and activation of the cell death pathway. **b** Activation of genes encoding homologs of an *Arabidopsis* $Ca^{2+}$ sensor and a $Ca^{2+}$ signaling pathway kinases involved in senescence regulation. Data are presented as mean values ± SD. For statistical analysis, count data at the gene level were analyzed with DESeq2[64]. Stage-to-stage comparisons were performed and corrected for multiple testing over all genes and cell stage comparisons using false discovery rate (FDR). Source data and adjusted *P* values are provided in the source data file. Letters above bars indicate significant differences (adjusted $P < 0.05$). Source data and adjusted *P* values are provided in the source data file.

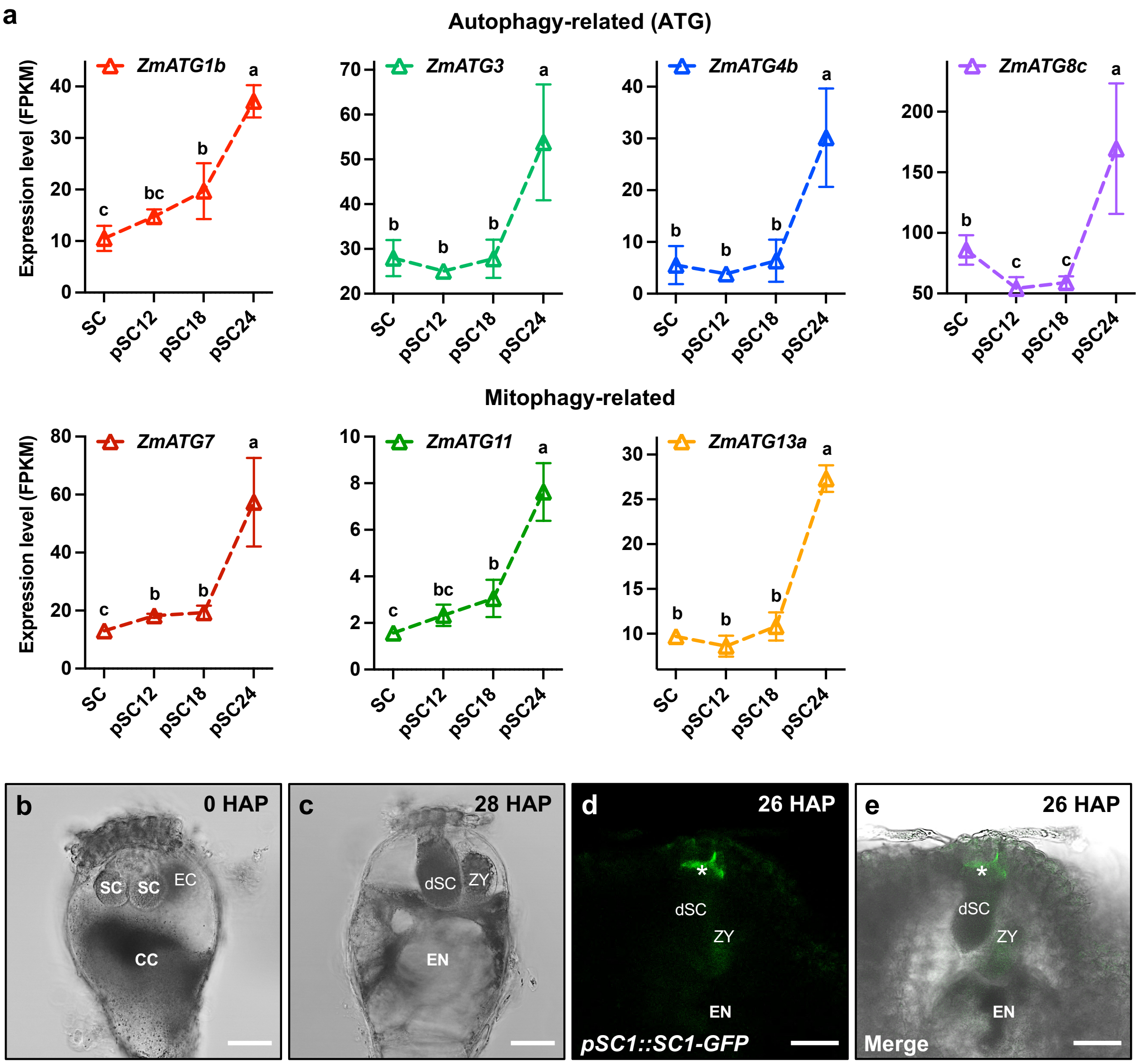


**Supplementary Fig. 12 Autophagy activation and subsequent elimination of the persistent synergid cell through synergid-endosperm (SE) fusion. a** Indicated general autophagy-related genes and mitophagy-related genes are strongly activated with delay after fertilization shortly before persistent synergid cell elimination. Data are presented as mean values ± SD. For statistical analysis, count data at the gene level were analyzed with DESeq2[64]. Stage-to-stage comparisons were performed and corrected for multiple testing over all genes and cell stage comparisons using false discovery rate (FDR). Source data and adjusted *P* values are provided in the source data file. Letters above bars indicate significant differences between developmental stages (adjusted $P < 0.05$). Source data and adjusted *P* values are provided in the source data file. **b–e** Persistent synergid cell elimination at the end of PCD culminating in synergid-endosperm (SE) fusion. Isolated maize embryo sac before pollination (b) and at 28 HAP (c) showing absence

of the persistent synergid cell and moderate plasmolysis of a syncytium at the micropylar region. Secreted SC1-GFP signals (asterisk) are only visible at the residual filiform apparatus of the eliminated persistent synergid cell at 26 HAP (d, e). Experiments were repeated three times with similar results and presented (in b–e) are representative images. Abbreviations: CC, central cell; dSC, degenerated receptive synergid cell; EC, egg cell; EN, endosperm; SC, synergid cell; ZY, zygote. Scale bars, 50 μm.
