# Supplementary material for: Fertilization-induced synergid cell death by RALF12-triggered ROS production and ethylene signaling
Source: Nat Commun. 2025 Mar 29;16:3059. doi: 10.1038/s41467-025-58246-y (PMC11953305; doi:10.1038/s41467-025-58246-y)
Supplement: Supplementary file 2 — Description of Additional Supplementary Files [file 41467_2025_58246_MOESM2_ESM.docx]

**Description of Additional Supplementary files**

Supplementary Data 1: Summary of cell- and stage-specific RNA-seq library statistics and read mapping results.

Supplementary Data 2: Expression level of each gene in each cell stage.

Supplementary Data 3: Subcellular distribution of most abundant gene products in different cells.

Supplementary Data 4: The most highly expressed genes in synergid cells encode especially synergid-specific/predominant secreted peptides and secreted cell wall modifiers.

Supplementary Data 5: Downregulation of most highly expressed genes after fertilization and during synergid degeneration.

Supplementary Data 6: The most strongly expressed TFs are synergid-specific and are substantially decreased after fertilization and during synergid degeneration.

Supplementary Data 7: Activation of ROS production, oxidative stress response, ethylene biosynthesis and signaling, and PCD regulation during synergid PCD.

Supplementary Data 8: The major transcriptional activation and repression wave occurs about 24 HAP (timing of successful fertilization).

Supplementary Data 9: *De nove* expressed genes all exhibit PCD-related expression patterns.

Supplementary Data 10: Primers used in the present study.
