# Supplementary material for: Fertilization-induced synergid cell death by RALF12-triggered ROS production and ethylene signaling
Source: Nat Commun. 2025 Mar 29;16:3059. doi: 10.1038/s41467-025-58246-y (PMC11953305; doi:10.1038/s41467-025-58246-y)
Supplement: Supplementary file 4 — Reporting Summary [file 41467_2025_58246_MOESM4_ESM.pdf]

nature portfolio

Corresponding author(s): Thomas Dresselhaus, Junyi Chen

Last updated by author(s): Feb 22, 2025

Reporting Summary

Nature Portfolio wishes to improve the reproducibility of the work that we publish. This form provides structure for consistency and transparency in reporting. For further information on Nature Portfolio policies, see our Editorial Policies and the Editorial Policy Checklist.

Statistics

For all statistical analyses, confirm that the following items are present in the figure legend, table legend, main text, or Methods section.

n/a	Confirmed	
☐	☒	The exact sample size (*n*) for each experimental group/condition, given as a discrete number and unit of measurement
☐	☒	A statement on whether measurements were taken from distinct samples or whether the same sample was measured repeatedly
☐	☒	The statistical test(s) used AND whether they are one- or two-sided<br>*Only common tests should be described solely by name; describe more complex techniques in the Methods section.*
☒	☐	A description of all covariates tested
☐	☒	A description of any assumptions or corrections, such as tests of normality and adjustment for multiple comparisons
☐	☒	A full description of the statistical parameters including central tendency (e.g. means) or other basic estimates (e.g. regression coefficient) AND variation (e.g. standard deviation) or associated estimates of uncertainty (e.g. confidence intervals)
☐	☒	For null hypothesis testing, the test statistic (e.g. F, t, r) with confidence intervals, effect sizes, degrees of freedom and P value noted<br>*Give P values as exact values whenever suitable.*
☒	☐	For Bayesian analysis, information on the choice of priors and Markov chain Monte Carlo settings
☒	☐	For hierarchical and complex designs, identification of the appropriate level for tests and full reporting of outcomes
☐	☒	Estimates of effect sizes (e.g. Cohen's *d*, Pearson's *r*), indicating how they were calculated

Our web collection on statistics for biologists contains articles on many of the points above.

Software and code

Policy information about availability of computer code

Data collection	RNA-seq data were generated from Illumina NovaSeq 6000 platform with PE150 mode. Microscopic images were acquired with the Leica application Suit X (ver. 3.7.6.25997).
Data analysis	Cutadapt (v1.15), Hisat2 (v2.0.5), featureCounts (v1.5.0-p3), DESeq2 were used for cell- and stage-specific RNA-seq data analysis. ImageJ (ver. 1.52a) was used to measure fluorescence intensity values. GraphPad Prism (v10.1.0) was used for general plots of data.

For manuscripts utilizing custom algorithms or software that are central to the research but not yet described in published literature, software must be made available to editors and reviewers. We strongly encourage code deposition in a community repository (e.g. GitHub). See the Nature Portfolio guidelines for submitting code & software for further information.

Data

Policy information about availability of data

All manuscripts must include a data availability statement. This statement should provide the following information, where applicable:

- Accession codes, unique identifiers, or web links for publicly available datasets
- A description of any restrictions on data availability
- For clinical datasets or third party data, please ensure that the statement adheres to our policy

All RNA-seq data have been uploaded to the NCBI Gene Expression Omnibus (GEO) under accession number GSE283809. Previously published RNA-seq data (GEO

accession number GSE98379) were also used in the present study. The core data of transcriptomics in the paper are also provided in the supplementary data file. Source data are provided with this paper.

Research involving human participants, their data, or biological material

Policy information about studies with human participants or human data. See also policy information about sex, gender (identity/presentation), and sexual orientation and race, ethnicity and racism.

Reporting on sex and gender	n/a
Reporting on race, ethnicity, or other socially relevant groupings	n/a
Population characteristics	n/a
Recruitment	n/a
Ethics oversight	n/a

Note that full information on the approval of the study protocol must also be provided in the manuscript.

Field-specific reporting

Please select the one below that is the best fit for your research. If you are not sure, read the appropriate sections before making your selection.

☒ Life sciences ☐ Behavioural & social sciences ☐ Ecological, evolutionary & environmental sciences

For a reference copy of the document with all sections, see nature.com/documents/nr-reporting-summary-flat.pdf

Life sciences study design

All studies must disclose on these points even when the disclosure is negative.

Sample size	Sample sizes were estimated based on our experiences in performing similar experiments and published works. Four different cell stages including synergid cell 0 HAP, persistent synergid cell 12 HAP, persistent synergid cell 18 HAP and persistent synergid cell 24 HAP were collected for RNA-seq. According to the common criteria, three independent biological replicates have been performed for each cell stage. RNA-seq data from three independent biological replicates of each cell stage are highly correlated (average R-squared = 0.93), and the transcripts of 14,700–15,800 genes were detected in each sample. The deviations between biological replicates are small and are thus highly reproducible and sufficient to draw solid conclusions. For other key data such as RALF peptides treatments, ethylene treatments and ROS scavenger application, at least three biological replicates were analyzed with a total sample number ranging from 28 (single embryo sacs) to up to 163 (single ovules). These sample sizes are enough for robust and reliable statistical analysis in the present study.
Data exclusions	No data were excluded from the analysis.
Replication	The transcriptome from three independent biological replicates within the same cell stage are highly correlated (average R-squared = 0.93). Each experiments was performed with at least two (mostly three) biological replicates, and only results representing the consistent outcome are reported.
Randomization	Three independent biological replicates were allocated into experimental groups according to the cell stage. All the experimental materials were randomly picked for test.
Blinding	Since three independent biological replicates for each cell stage were already allocated into the same group, blinding was not relevant to our study. For comparative analyses, plants were grown under the same conditions and control experiments were always performed in parallel.

Reporting for specific materials, systems and methods

We require information from authors about some types of materials, experimental systems and methods used in many studies. Here, indicate whether each material, system or method listed is relevant to your study. If you are not sure if a list item applies to your research, read the appropriate section before selecting a response.

Materials & experimental systems

n/a	Involved in the study
☐	☒ Antibodies
☒	☐ Eukaryotic cell lines
☒	☐ Palaeontology and archaeology
☒	☐ Animals and other organisms
☒	☐ Clinical data
☒	☐ Dual use research of concern
☐	☒ Plants

Methods

n/a	Involved in the study
☒	☐ ChIP-seq
☒	☐ Flow cytometry
☒	☐ MRI-based neuroimaging

Antibodies

Antibodies used

ZmRALF12 antibody (Generated by Proteingene Biotech), 1:200 dilution
Dylight 488-conjugated secondary antibody (Abbkine; Catalog number: A23220), 1:400 dilution

Validation

ZmRALF12 antibody was generated by Proteingene Biotech (http://www.proteingene.com/). Dylight 488-conjugated secondary antibody was bought from Abbkine (https://www.abbkine.com/product/dylight-488-goat-anti-rabbit-igg-a23220/).

Plants

Seed stocks

Maize (Zea mays) inbred line B73 and the synergid marker line pZmES4::ZmES4-GFP seeds were preserved in our laboratory.

Novel plant genotypes

pZmSC1::ZmSC1-GFP and pZmSC3::ZmSC3-GFP transgenic lines and zmralf12 mutants (by CRISPR-Cas9 technique) were generated in the B104 background via Agrobacterium tumefaciens-mediated transformation.

Authentication

The mutations in ZmRALF12 genome sequence were determined by Sanger sequencing on PCR products that covered the target sites. We mainly focus on the phenotypes during persistent synergid degeneration, ZmRALF12 is the only gene in the maize RALF gene family that is highly induced in this process. Additionally, we observed the phenotype with different independent zmralf12 lines. These can effectively exclude potential off-target effects. pZmES4::ZmES4-GFP, pZmSC1::ZmSC1-GFP and pZmSC3::ZmSC3-GFP transgenic lines were confirmed by PCR. At least two transgenic lines for each construct were used for fluorescent signal observation and microscopy imaging.
